# Characterization of the input material quality for the production of tisagenlecleucel by multiparameter flow cytometry and its relation to the clinical outcome

**DOI:** 10.3389/pore.2023.1610914

**Published:** 2023-04-20

**Authors:** Martin Štach, Robert Pytlík, Kristýna Šmilauerová, Jana Rychlá, Martin Mucha, Jan Musil, Abhishek Koladiya, Matěj Nemec, Martina Petráčková, Iva Kaštánková, Pavla Pecherková, Lucie Šrámková, Kamila Polgárová, Marek Trněný, Petr Lesný, Jan Vydra, Pavel Otáhal

**Affiliations:** ^1^ Institute of Hematology and Blood Transfusion, Praha, Czechia; ^2^ Faculty of Science, Charles University, Praha, Czechia; ^3^ First Faculty of Medicine, Charles University, Praha, Czechia; ^4^ Second Faculty of Medicine (2. LF UK), University Hospital in Motol, Praha, Czechia; ^5^ General University Hospital in Prague, Praha, Czechia

**Keywords:** immunotherapy, CAR-T cells, tisagenlecleucel, B-cell lymphoma and leukemia, Kymriah

## Abstract

Tisagenlecleucel (tisa-cel) is a CD19^-^specific CAR-T cell product approved for the treatment of relapsed/refractory (r/r) DLBCL or B-ALL. We have followed a group of patients diagnosed with childhood B-ALL (*n* = 5), adult B-ALL (*n* = 2), and DLBCL (*n* = 25) who were treated with tisa-cel under non-clinical trial conditions. The goal was to determine how the intensive pretreatment of patients affects the produced CAR-T cells, their *in vivo* expansion, and the outcome of the therapy. Multiparametric flow cytometry was used to analyze the material used for manufacturing CAR-T cells (apheresis), the CAR-T cell product itself, and blood samples obtained at three timepoints after administration. We present the analysis of memory phenotype of CD4/CD8 CAR-T lymphocytes (CD45RA, CD62L, CD27, CD28) and the expression of inhibitory receptors (PD-1, TIGIT). In addition, we show its relation to the patients’ clinical characteristics, such as tumor burden and sensitivity to prior therapies. Patients who responded to therapy had a higher percentage of CD8^+^CD45RA^+^CD27^+^ T cells in the apheresis, although not in the produced CAR-Ts. Patients with primary refractory aggressive B-cell lymphomas had the poorest outcomes which was characterized by undetectable CAR-T cell expansion *in vivo*. No clear correlation of the outcome with the immunophenotypes of CAR-Ts was observed. Our results suggest that an important parameter predicting therapy efficacy is CAR-Ts’ level of expansion *in vivo* but not the immunophenotype. After CAR-T cells’ administration, measurements at several timepoints accurately detect their proliferation intensity *in vivo*. The outcome of CAR-T cell therapy largely depends on biological characteristics of the tumors rather than on the immunophenotype of produced CAR-Ts.

## Introduction

Tisagenlecleucel (tisa-cel, Kymriah^®^) is CD19^-^specific CAR-T-cell product approved for the treatment of relapsed/refractory (r/r) DLBCL or B-ALL. First, an input material is obtained *via* apheresis from individual patients to produce CAR-T cells. Collected peripheral blood mononuclear cells (PBMCs) are then cryopreserved and supplied to the manufacturer. Followingly, PBMCs are transduced with lentiviral vector, expanded *in vitro*, and cryopreserved. The generated CAR-T cells are usually within a month supplied back to the hospital site. However, such a highly complex process utilizes materials obtained from patients who received multiple lines of intensive chemotherapeutic regimens and have active disease. Additionally, the variability within differentiation/memory subsets of T cells in the apheresis might affect the quality of produced CAR-T cells and subsequently limit the efficacy of the treatment (1, 2).

Treatment of DLBCL with CD19^-^specific CAR-T cells such as tisa-cel is slightly less effective than identical approach to patients with B-ALL (2–6). Generally, two types of treatment failure are encountered. The first type is non-responsiveness—the patients do not achieve even a partial remission (PR) within 2–3 months post-treatment. The second type is late relapse after achievement of good clinical response following CAR-T cell treatment (7). Detailed analysis of the input material and the produced CAR-T cells could help identify factors responsible for these types of treatment failures (8–10).

The first goal of our study was to perform a detailed FACS analysis of the apheretic material used for CAR-T cells’ production to determine present leukocyte subsets and the immunophenotype of T cells. Secondly, the manufactured product was analyzed to determine the percentage of CAR^+^ cells and their memory phenotype. Thirdly, CAR-T cells’ expansion kinetics and their differentiation status were measured in samples of peripheral blood after treatment. Collected measurements were compared with the treatment efficiency and patients’ survival.

In summary, this study describes the analysis of apheresis, manufactured CAR-T cells and samples of patients treated with tisa-cel. Based on obtained measurements and clinical data, our results suggest that a specific phenotype of starting material (i.e., apheresis) influences possible success of the therapy. The results also show that undetectable CAR-T cell expansion at D+14 is linked to early treatment failure (*p* < 0.05) which might provide a chance to effectively indicate the patient enrollment into clinical trials.

## Results

### Detection of CAR-T cells and gating strategy for their phenotyping

**FIGURE 1:**
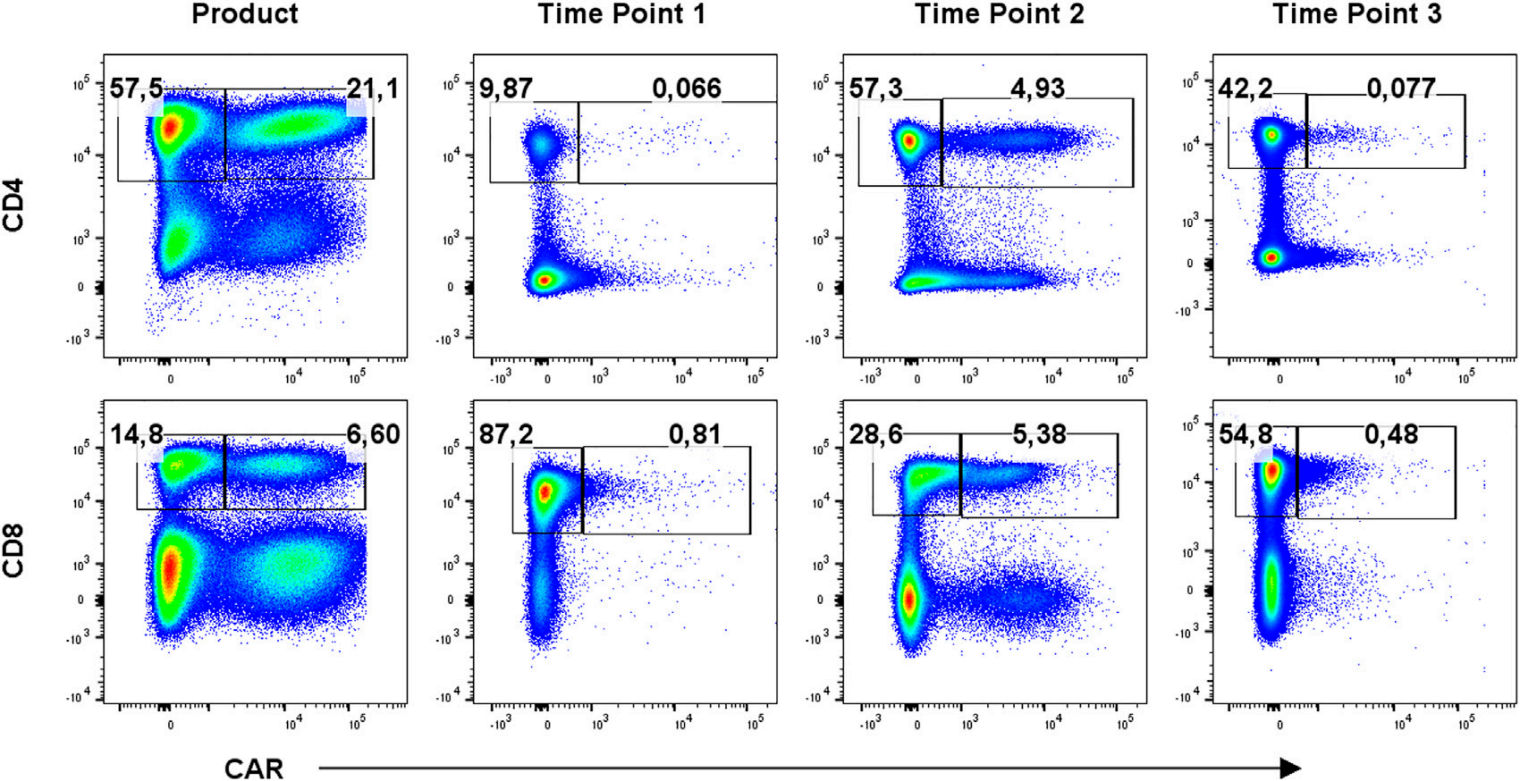
Detection of CAR-T cells in the product and in blood at three timepoints: T1 early after infusion (day 2–4), T2 at the expected peak of expansion (days 10–14), and T3 at the predicted contraction phase (days 30–60). Percentages of CAR^−^ (left gate) and CAR^+^ (right gate) cells of CD3^+^ cells are shown for one representative patient. Gates were set based on measurements of healthy donor control.

**FIGURE 2:**
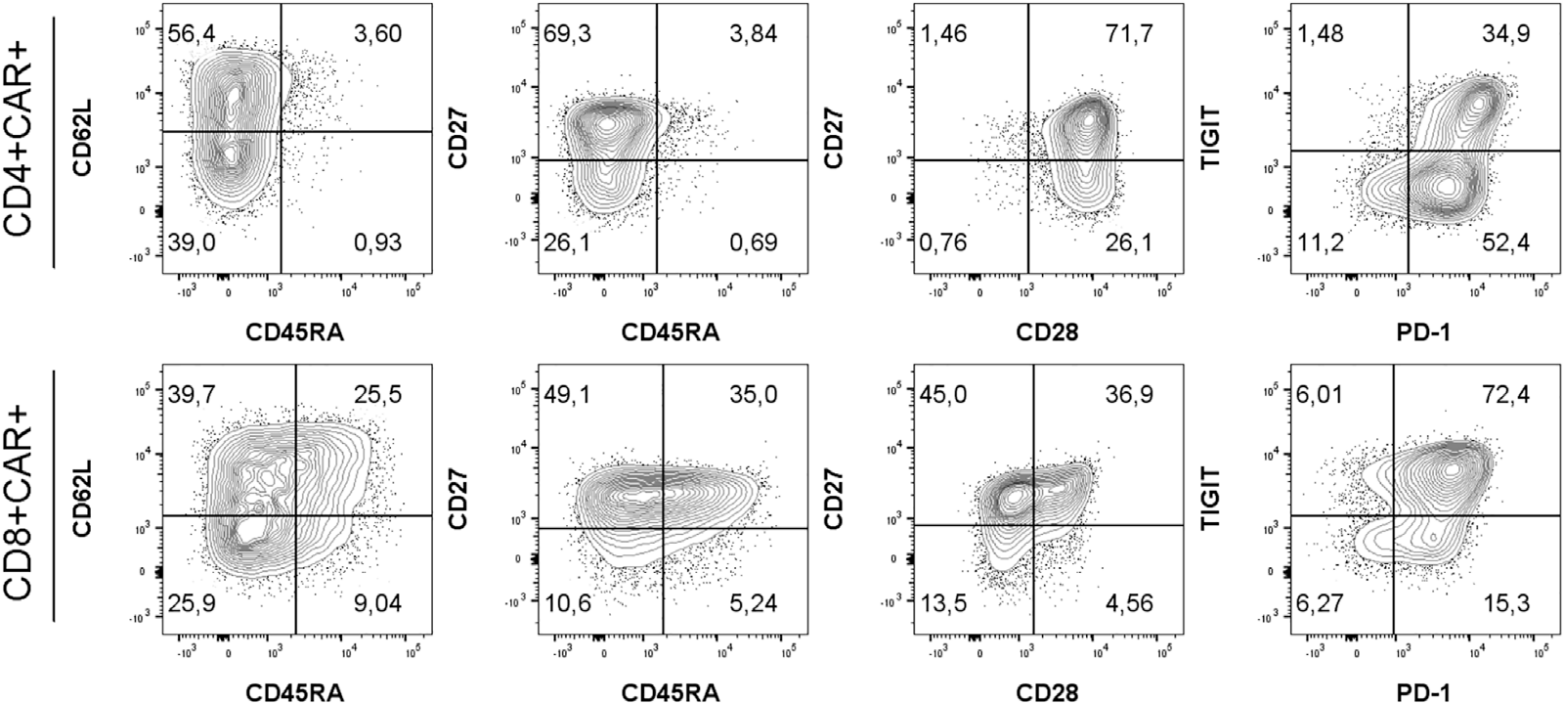
Gating strategy for immunophenotyping of CD4^+^/CD8^+^ CAR-T cells using antibodies against antigens CD45RA, CD62L, CD27, CD28, PD-1 and TIGIT. Memory subsets were defined as: SCM, stem cell memory (CD45RA^+^CD62L^+^); CM, central memory (CD45RA^−^CD62L^+^); EM, effector memory (CD45RA^−^CD62L^−^); EMRA, terminally differentiated effector memory (CD45RA^+^CD62L^−^). One representative patient is shown. Percentages of populations of CD4^+^CAR^+^ or CD8^+^CAR^+^ shown in respective quadrants. Gates were set based on measurements of healthy donor control and FMO controls.

To identify CAR-T cells, we used recombinant PE-conjugated CD19 protein for the first eight patients due to the unavailability of anti-FMC63 antibody, which was then used for the remaining patients. Example of staining is presented in Supplementary Figure S1 *.* CAR-T cells were further stained with a multicolor antibody panel to determine their differentiation immunophenotype using antibodies against antigens CD3, CD4, CD8, CD14, CD45RA, CD62L, CD27, CD28, PD-1, and TIGIT (11). Additionally, samples were analyzed with a second antibody panel to determine the composition of all significant leukocyte subsets using antibodies against antigens CD3, CD4, CD8, CD14, CD16, CD19, CD45, CD56, and TCRgd. Figure 1 shows an example of typical CAR detection. Gating strategy of CAR-T memory subsets is similarly presented in Figure 2. Pre-gating on CD3^+^ cells is shown in Supplementary Figure S2. Controls for CAR staining (healthy donor) and immunophenotype staining (FMOs, healthy donor) are presented in Supplementary Figures S3, S4, respectively. Threshold for the detection of CAR^+^ cells was set at 0.1% out of CD3 T cells, and the percentage of CAR-T cells was further converted to the absolute counts per microliter using the total white blood cell count values obtained from hematology analyzer. The antibody panels are presented in Supplementary Table S1 *.*

### Analysis of apheresis and CAR-T cell products

**FIGURE 3:**
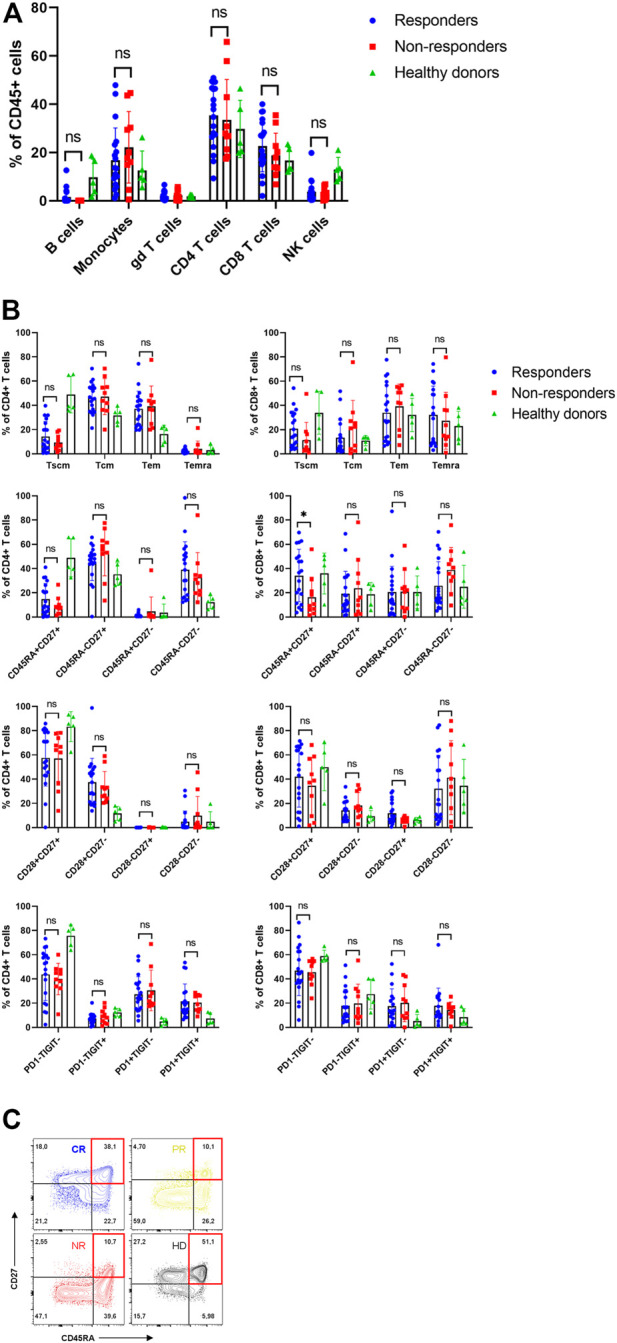
Analysis of apheresis. **(A)** Composition of individual leukocyte subsets in the apheresis. **(B)** Immunophenotype of CD4^+^ and CD8^+^ T cells in apheresis of responders, non-responders and healthy donors. SCM, stem cell memory; CM, central memory; EM, effector memory; EMRA, terminally differentiated effector memory. **(C)** Representative immunophenotypes of T cells (CD8^+^CD27^+^DC45RA^+^) as percentages of CD8^+^ cells in four subjects: CR, complete response; PR, partial response; NR, non-responder; HD, healthy donor. Unpaired *t*-test *p < 0.05, ns-not significant.

The first goal was to characterize patients’ apheresis and to determine whether differences in their T cell memory subsets are associated with stronger *in vivo* expansion of CAR-T cells and improved clinical outcome. For each patient, the apheretic product samples were cryopreserved, as well as from five healthy donors functioning as control samples. Prior to flow cytometry analysis, the cells were thawed and let to rest in media overnight. We have found out that 27 out of 32 subjects had undetectable B-cells in apheresis due to preceding treatment by rituximab (Figure 3A, Supplementary Figure S2). To characterize the T cells, we determined their immunophenotype with the differentiation antibody panel (omitting the anti-FMC63 antibody). The patients’ T cells were characterized by highly variable numbers of Tem/Tcm/Temra CD8^+^ and CD4^+^ memory subsets, similarly to what was detected in healthy donors (Figure 3B, Supplementary Figure S6). Nevertheless, the patients’ T cells contained more exhausted T cells expressing PD-1 receptor compared to healthy donors—50% vs. 12% CD4^+^ (*p* = <0.001) and 35% vs. 14% CD8^+^ (*p* = 0.046). In addition, the non-responders were characterized by significantly reduced numbers of CD8^+^ CD45RA^+^ CD27^+^ T cells (Figure 3C) in the apheresis, in agreement with report by Fraietta et al. (12).

**FIGURE 4:**
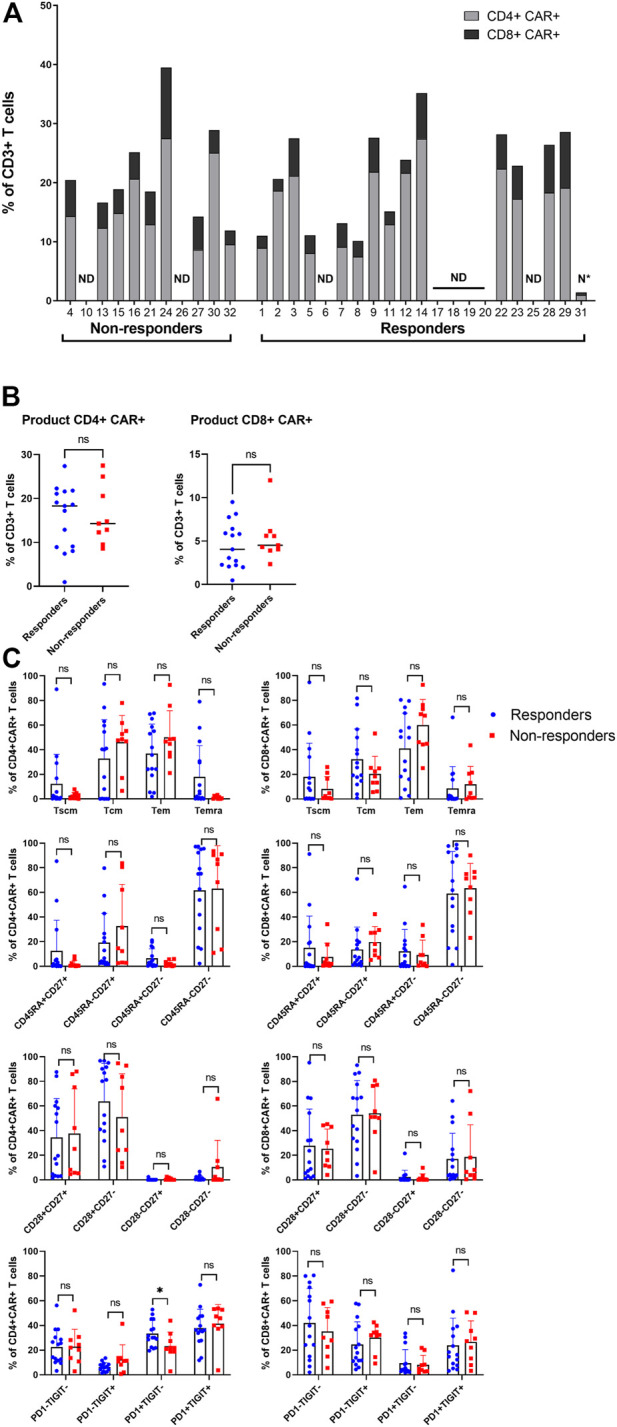
Analysis of the product. **(A)** Manufactured products cells were analyzed to determine the percentage of CAR^+^ in CD4^+^ and CD8^+^ T cells out of all CD3^+^ T cells. ND, not done; N*, product not meeting specifications. **(B)** Statistical analysis of the percentages of CD4^+^CAR^+^ (left) and CD8^+^CAR^+^ (right) in product of responders and non-responders. **(C)** Immunophenotypes of CD4^+^CAR^+^ and CD8^+^CAR^+^ cells in product of responders and non-responders. SCM, stem cell memory; CM, central memory; EM, effector memory; EMRA, terminally differentiated effector memory. Unpaired *t*-test; ns, not significant, *p < 0.05.

Subsequently, the manufactured CAR-T cells were analyzed using aforementioned antibody panel with added anti-FMC63 antibody. The samples for measurements were obtained from discarded infusion bags after administration to the patients. The infusion bags and their filters were thoroughly washed with PBS to acquire the remaining cells. In some cases, we were unable to measure administered products for technical reasons. We observed that the products contained a highly variable percentage of CAR^+^ cells (range 1%–40%) and that the majority of CAR-T cells were CD4 positive (Figure 4A). There was no significant difference of percentage of CD4^+^ or CD8^+^ CAR-T cells in product between responders and non-responders (Figure 4B). The immunophenotype of CAR-T cells was likewise highly variable between individual patients in regards to the proportion of Tscm/Tcm/Tem/Temra, and CD27/CD28/PD-1/TIGIT positive cells (Figure 4C, Supplementary Figure S7). There was a slightly higher PD1+TIGIT− percentage of CD4^+^CAR^+^ T cells in responders (*p* = 0.039, Figure 4C). CAR^+^ T cells in the product had almost identical immunophenotype as the CAR^−^ population (data not shown), suggesting that their differentiation during manufacturing was driven more by polyclonal anti-CD3/CD28 activation than by CAR signaling. One product (P31) did not meet the entry specifications since it contained only 1% of CAR-T cells. Despite such a low percentage of CAR-T cells, this patient responded to the therapy and achieved PR by PET/CT (nonetheless relapsed later). Conversely to apheresis, we did not detect a significant difference in the numbers of CD8^+^ CD45RA^+^ CD27^+^ T cells in the product (Figure 4C).

### Fate of CAR-T cells *in vivo*

**FIGURE 5:**
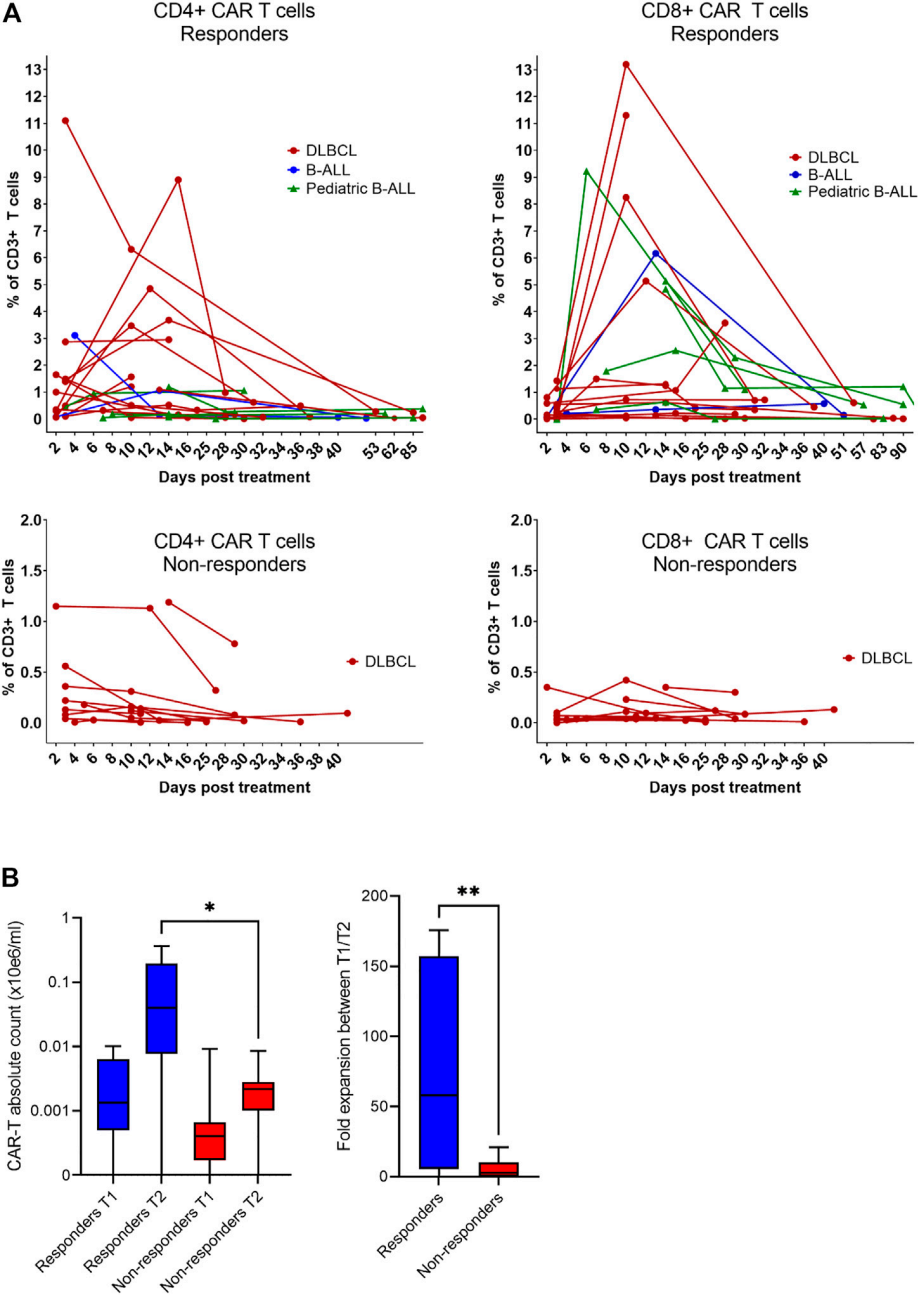
Expansion of CAR-T cells *in vivo*. **(A)** The level of expansion of CAR-T cells was determined in peripheral blood samples at three timepoints after administration: early after infusion (day 2–4), at the expected peak of expansion (days 10–14), and at the predicted contraction phase (days 30–60). The left panels show the expansion kinetics of CD4^+^CAR^+^ cells and right panels shows CD8^+^CAR^+^ cells as a percentage of CD3^+^ T cells. **(B)** Statistical analysis of the level of expansion in responders (*n* = 13) vs. non-responders (*n* = 10) at indicated timepoints shown as a total number of CAR-T cells in the blood and as a fold expansion of absolute CAR-T cell counts in the blood between timepoint 1 (T1) and timepoint 2 (T2). Unpaired *t*-test *p < 0.05, **p < 0.005.

**FIGURE 6:**
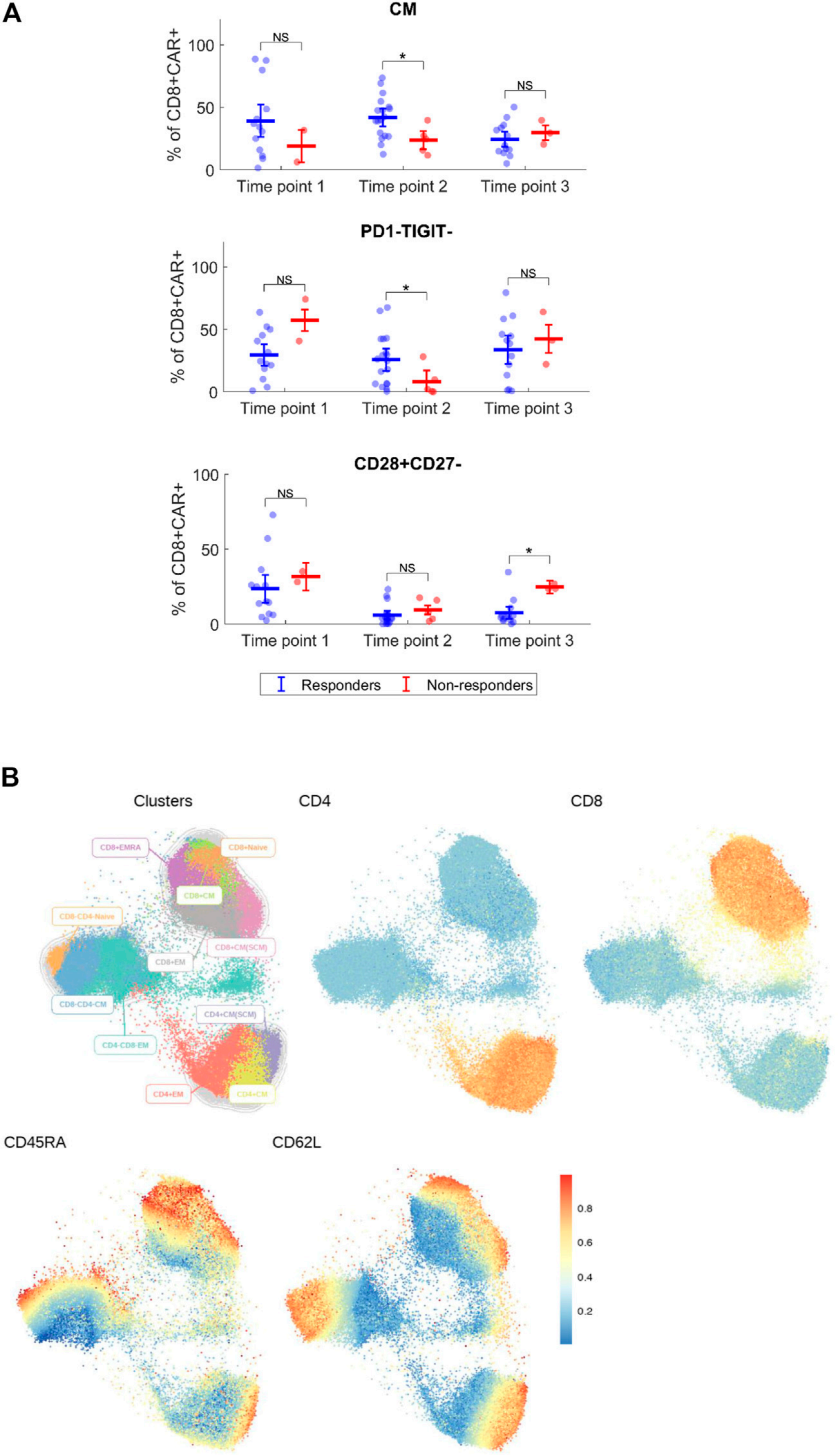
Immunophenotype of CAR-T cells *in vivo*. **(A)** Expanded CD4^+^ and CD8^+^ CAR^+^ T cells were analyzed for each patient by multiparameter flow cytometry to determine the expression of major surface antigens reflecting their differentiation status. Phenotypes with significant difference between responders and non-responders are shown. Percentages of phenotype subsets are of CD8^+^ CAR^+^ T cells. CM, central memory (CD45RA^−^CD62L^+^). Mann-Whitney test; ns, not significant, *p < 0.05. **(B)** Cluster analysis of detected CAR^+^ T cells at the first timepoint for indicated antigens, all identified CAR-T cells from all responders and non-responders at the first timepoint were analyzed together. EmbedSOM algorithm was used. The scale indicates relative intensity of detected antigens.

Expansion and immunophenotype of CAR-T cells were determined in the patients’ blood samples after administration at three timepoints—between days 2–4 (T1), days 10–14 (T2), and days 30–60 (T3). Since the study was performed in a non-clinical trial setting, the samples could be obtained only during regular medical examinations including signing of the informed consent form by the patient. To determine the kinetics of CAR-T cells’ expansion, we measured percentage of CD4^+^/CD8^+^ CAR-T out of CD3^+^ T cells and calculated their absolute counts in blood. The CAR-T cell percentages were compared between T1 and T2 to assess CAR-T cells’ expansion kinetics. If the percentage of either CD4^+^ or CD8^+^ CAR^+^ at T2 was higher than their percentage at T1, the patient was categorized as showing a detectable expansion. Figure 5A shows the kinetics of CAR-T cell expansion for each patient. In addition, we calculated the absolute number of CAR-T cells per µl of blood in the responders and the non-responders at T1 and T2 (Figure 5B). Table 1 shows the measured values of CAR-T cells in blood for each patient and demonstrates that only 27% (3/11) of non-responders had a detectable expansion of CAR-T cells and that 90% (19/21) of responders had detectable expansion (Table 2). Out of 25 patients with DLBCL, 10 subjects had no expansion of CAR-T cells (all patients with B-ALL were responders with detectable CAR-T cell expansion). 80% of these “non-expanders” were also non-responders, but only 40% of them had primary refractory disease. Following this, we determined the immunophenotype of detected CAR-T cells in the blood similarly as was done for the product (Supplementary Figure S8). Threshold for the detection of CAR-T cells was set at 0.1% of CD3^+^ T cells, samples with CAR^+^ percentage below this value were not analyzed. Significant differences between responders and non-responders are presented in Figure 6A. At time point 2, responders had higher numbers of CD8^+^CAR^+^ Tcm (*p* = 0.030) and PD1−TIGIT− than non-responders (*p* = 0.048). At time point 3, CD28^+^CD27^−^ percentage was higher in non-responders (*p* = 0.025), although only 3 samples were used for the analysis. Clustering by EmbedSOM algorithm (13) shows relative distribution of memory subsets of CAR-T cells for time point 1 (Figure 6B).

**TABLE 1:** Patients’ characteristics.

Patient ID	Age	Dg	Primary refractory dissease	Sensitivity to bridge treatment before CAR-T	Clinical stage before CAR-T	Response to CAR-T at 3 m/6 m	Lymphodepletion regimen	Number of lines of treatment before CAR-T	T1 %CD4^+^CAR^+^	T2% CD4	T1 %CD8		T2 %CD8	CAR-T expansion
1	25	ALL	No	Yes	CR	CR/CR	Flu/Cy	3+alloSCT	0	1.06	0		6.17	Yes
2	27	ALL	No	Yes	CR	CR/relapse	Flu/Cy	5+alloSCT	3.11	0.15	0.21		0.36	Yes
3	69	DLBCL	No	Yes	CR	CR/CR	Flu/Cy	4+ASCT	0	4.85	0.81		5.14	Yes
4	67	DLBCL	No	Yes	2	progression	None	4+ASCT	1.15	1.13	0.35		0	No
5	43	DLBCL	No	Yes	1	CR/CR	Flu/Cy	4	1.39	3.47	0.4		8.25	Yes
6	53	DLBCL	Yes	No	4	PR/CR	bendamustine	4	0.35	0.16	0		0.22	Yes
7	74	DLBCL	No	Yes	1	CR/CR	Flu/Cy	5+ASCT	2.87	2.95	1.13		1.32	Yes
8	77	DLBCL	No	Yes	4	CR/CR	Flu/Cy	3	1.46	3.68	0.55		0.62	Yes
9	34	DLBCL	No	No	4	PR/progression	Flu/Cy	3+ASCT	0.3	8.9	0.59		1.07	Yes
10	61	DLBCL	Yes	No	4 (bulky)	progression	Flu/Cy	4	ND	1.19	ND		0.35	Yes
11	72	DLBCL	No	Yes	CR	CR/CR	bendamustine	3+ASCT	ND	1.1	ND		0.14	Yes
12	39	DLBCL	No	Yes	2	CR/CR	Flu/Cy	3+ASCT	1	0	0.16		0	No
13	57	DLBCL	Yes	No	4	progression	bendamustine	4	0	0	0		0	No
14	41	FL/DLBCL	No	Yes	4	CR/CR	bendamustine	3+ASCT	1.65	0	0		0	No
15	54	DLBCL	No	No	3	progression	bendamustine	3+ASCT	0.56	0	0		0	No
16	49	DLBCL	No	Yes	4	progression	bendamustine	2	0.18	0	0		0.11	No
17	10	ALL	No	Yes	4	CR/CR	Flu/Cy	1	ND	1.19	ND		4.84	Yes
18	15	ALL	No	Yes	2	CR/CR	Flu/Cy	1	0.18	0.14	1.79		2.56	Yes
19	5	ALL	No	Yes	1	CR/CR	Flu/Cy	1	0	0.18	0.36		0.64	Yes
20	3	ALL	No	Yes	2	CR/CR	Flu/Cy	1	ND	0	ND		5.14	Yes
21	74	DLBCL	Yes	No	4	progression	Flu/Cy	3	0.10	0.12	0		0.23	Yes
22	72	DLBCL	No	No	2	CR/CR	bendamustine	3	11.10	6.31	1.43		13.20	Yes
23	71	FL/DLBCL	No	Yes	1	CR/CR	bendamustine	4	1.49	0.51	0.27		0.73	Yes
24	70	DLBCL	Yes	Yes	2	progression	bendamustine	2	0.22	0.14	0		0	No
25	10	ALL	No	Yes	PR	CR/CR	Flu/Cy	1	0.45	0.95	0		9.23	Yes
26	44	DLBCL	Yes	No	4	progression	None	2	0	0	0		0	No
27	76	DLBCL	No	No	4	progression	bendamustine	2	0.36	0.31	0.10		0.42	Yes
28	23	DLBCL	No	Yes	4	PR/---	pixantron+ benda	5	0.12	1.57	0		11.30	Yes
29	49	DLBCL	No	Yes	4	CR/CR	Flu/Cy	4+ASCT	0.49	1.20	0.22		0.12	Yes
30	64	DLBCL	Yes	No	4 (bulky)	progression	Flu/Cy	3	0.13	0	0		0	No
31	56	DLBCL	No	Yes	4 (bulky)	PR/progression	Flu/Cy	3	0.10	0.32	0.14		1.50	Yes
32	70	FL/DLBCL	No	Yes	3	progression	Flu/Cy	3	0	0.16	0		0	No

**TABLE 2:** Characteristics of DLBCL patients.

	Expansion of CAR-T	Primary refractory disease	Response to bridge treatment before CAR-T	Median clinical stage before CAR-T
Responders	87.5% (12/14)	7.1% (1/14)	78.5% (11/14)	3
Non-responders	27.3% (3/11)	54.5% (6/11)	36.4% (4/11)	4

### Non-blood samples and second dose administration

**FIGURE 7:**
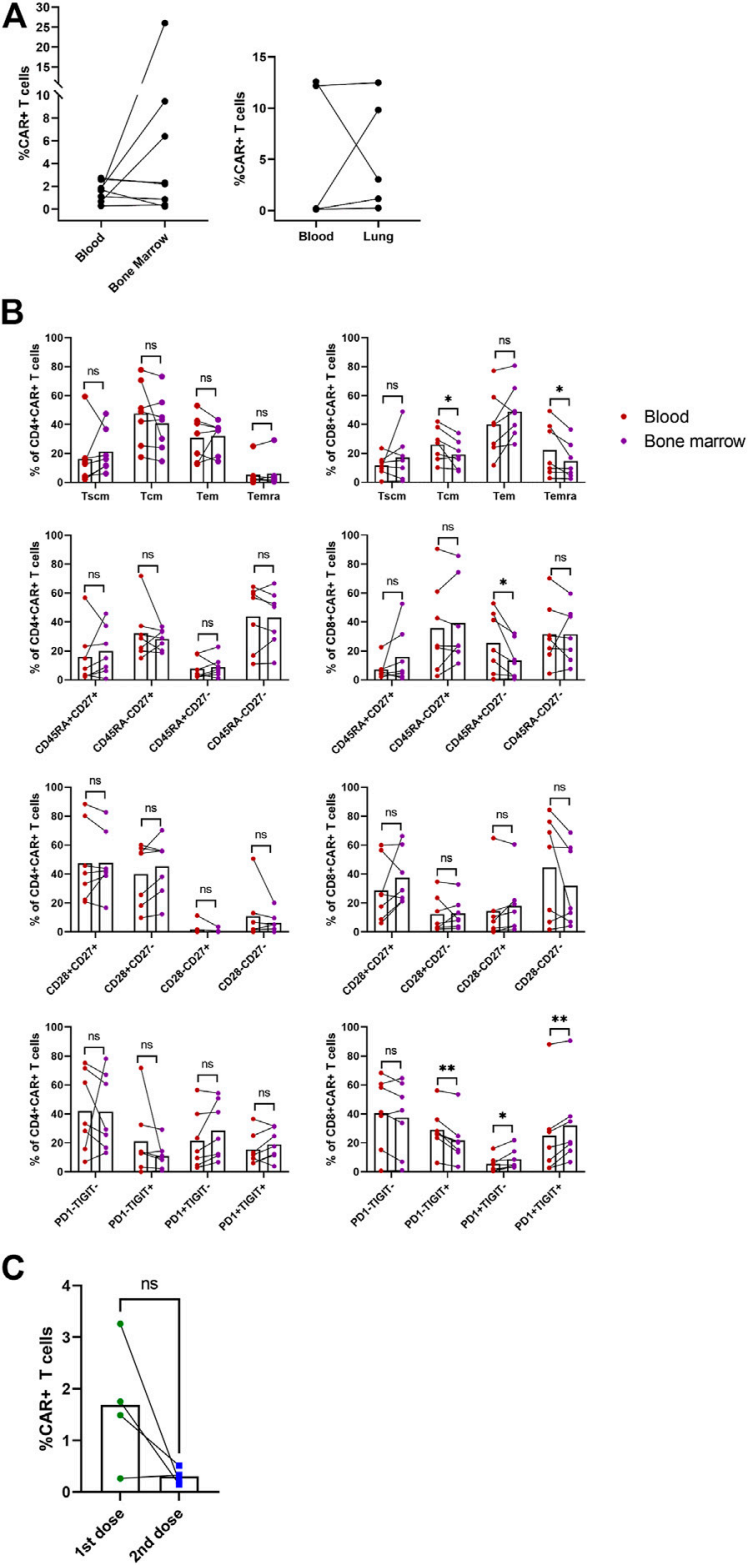
Non-blood samples and second dose administration. The indicated samples were analyzed by multiparametric flow cytometry to determine the percentage and immunophenotype of CAR-T cells. **(A)** Paired percentages of CD3^+^CAR^+^ cells in blood/bone marrow or blood/fluidothorax (lung). **(B)** Comparison of phenotypes of CAR^+^ cells in blood and bone marrow. SCM, stem cell memory; CM, central memory; EM, effector memory; EMRA, terminally differentiated effector memory. Paired *t*-test; ns, not significant, *p < 0.05, **p < 0.005. **(C)** Percentages of CAR-T cells in blood for the first and second dose at T2—D+14. Paired *t*-test; ns, not significant.

In addition to blood samples, we analyzed samples obtained from bone marrow and in three subjects with lung infiltration also from the malignant pleural effusion. Figure 7A shows percentages of CAR-T cells detected at a single time point in blood with corresponding bone marrow or pleural effusion (lung) sample. Interestingly, we observed effective migration of CAR-T cells into the tumor sites, demonstrated by high CAR-T percentage in the pleural effusion. We also detected long-term persisting CAR-T cells in the bone marrow of several subjects.

Immunophenotype of CAR-T cells in bone marrow was compared to paired blood samples (Figure 7B). CD8^+^ CAR-T cells in blood had elevated Tcm (*p* = 0.038), Temra (*p* = 0.046), and CD45RA^+^CD27^−^ (*p* = 0.032) phenotypes compared to bone marrow. CD8^+^ CAR-T cells in bone marrow had slightly higher percentage of PD1+TIGIT+ (*p* = 0.003), PD1+TIGIT− (*p* = 0.028) populations, and lower PD1−TIGIT+ (*p* = 0.004) percentage. Phenotype of CAR-T cells in pleural effusion was not statistically analyzed due to insufficient number of paired blood samples with detectable CAR-T cells (Supplementary Figure S9).

Four patients from the selected study group were administered a second dose of CAR-T cells after they relapsed; the source of the CAR-T cells was a remaining cryopreserved bag of the original product. Subject P09 received the second dose simultaneously with anti-PD-1 antibody pembrolizumab. Subject P16 received the second dose concurrently with lenalidomide treatment. Subjects P18 and P19 were pediatric B-ALL patients in clinical remission, although B-cell presence in the peripheral blood was established, suggesting the therapy’s failure. Therefore, the second CAR-T cells’ dose was used successfully as a bridging therapy before allo-HSCT. Furthermore, we observed that the subjects P16, P18, and P19 had a non-detectable expansion of the second CAR-T cells’ dose. In contrast, in the subject P09, we observed high levels of CAR-T cells in the pleural effusion even though they were almost undetectable in the blood sample. Figure 7C shows percentage of CAR-T cells at D+14 for first and second administration. The immunophenotype of these CAR-T cells was very similar to the immunophenotype of CAR-T cells detected in the patient’s blood sample following the administration of the first dose. Unfortunately, despite such a very efficient expansion of CAR-T cells, subject P09 did not achieve complete remission (CR) and the disease continually progressed.

## Discussion

In this report, we analyzed a group of 32 patients diagnosed with either B-ALL or DLBCL and treated in a real-world setting with tisa-cel, CD19^-^specific CAR-T cell product. The high costs of commercial CAR-T cell therapy and extended production time emphasize the need to search for clinical and laboratory parameters that will enable selection of patients with the highest chances to respond to therapy and determine what prior therapies might adversely affect the efficiency of the production of CAR-T cells. Thus, the main goals of our study were to correlate the efficacy of the therapy with the immunophenotype of input material used for the production of CAR-T cells (i.e., the apheresis) and with the immunophenotype of the produced CAR-T cells. Additionally, we observed CAR-T cell expansion kinetics after administration in the blood and measured their immunophenotype. The analysis presents a comprehensive data set from 32 subjects, including detailed clinical parameters such as prior treatments and complications associated with CAR-T cell therapy.

We observed a similar efficiency of the treatment in comparison to reported results of other real-world experiences (13). A noticeable subset of patients were non-responders who were characterized by continuously progressing disease after CAR-T cell therapy without achieving even a PR at 3-month restaging by PET/CT or by flow cytometry. In all cases, these patients were diagnosed with DLBCL and have shown no detectable expansion of CAR-T cells. Unfortunately, several patients with promising CR relapsed at later time points. The highly variable numbers and ratios of CD4^+^ and CD8^+^ T cells and the depletion of central memory (Tcm)/stem cell memory T cell (Tscm) subsets due to prior chemotherapies is a challenging issue for cellular therapy approaches. These subsets are essential for durable anti-tumor responses in adoptive cell therapy (14). For example, B-cell lymphoma patients have an increased percentage of terminal effector memory CD8^+^ T cells (15). Comparably to published reports (12), we found that an increased number of stem-cell-like memory CD45RA^+^CD27^+^ CD8^+^ T cells in the apheresis correlated with enhanced efficacy of CAR-T therapy, suggesting that the quality of the input material influences outcome of the treatment. However, the responders did not have a significantly higher number of CD45RA^+^CD27^+^ CAR-T cells in the product in comparison to the non-responders.

Next, the product analysis showed an interesting, highly variable CD4/CD8 ratio of CAR-T cells between products. Moreover, the percentage of CAR^+^ T cells in the product did not correlate with the response to the treatment suggesting that out-of-specification products might be equally efficient and worth administering (Figure 4B). For example, patient 32 had a bulky tumor and achieved near CR despite the administered product containing only 2% of CAR^+^ cells. These findings indicate that an efficient response probably requires a relatively small number of CAR^+^ cells with stem-cell memory phenotype, which can be reliably achieved in most patients despite significant differences in the T cell subsets between products.

Immunophenotypic analysis of expanded CAR-T cells *in vivo* revealed higher numbers of central memory and PD1−TIGIT− CD8^+^CAR^+^ cells in responders at time point 2 (Figure 6A). This might contribute to the hypothesis that early memory phenotypes are persisting longer, and thus leading to a better response. However, more data is needed to further support these findings.

Performed studies analyzing factors associated with responsiveness to CAR-T cell therapy among patients with DLBCL showed that one of the most significant negative factors is tumor mass volume (5, 7). We similarly observed the lowest efficacy of the treatment among patients with primary refractory DLBCL who frequently were at clinical stage IV with a large tumor burden. Furthermore, these patients continuously undergo intensive chemotherapy, negatively impacting CAR-T cells’ quality. Thus, a combination of these factors might severely impair the treatment outcome among patients with primary refractory DLBCL. Any solution to these problems would be complicated − for example, such patients could be considered for clinical trials with enhanced next-generation CAR-T cell products, which can be rapidly manufactured from the original apheresis for commercial CAR-T cell products (16). Hence, patients with primary refractory DLBCL without detectable CAR-T cell expansion would be such candidates. Therapeutic options for relapsed patients after CAR-T cell therapy are purely experimental; a frequently used option is the second dose of CAR-T cell infusion (17). We have analyzed four of these subjects. However, we observed, similarly to already reported data, a significantly reduced expansion of CAR-T cells compared to the first treatment except for subject P09 (who also received an anti-PD1 antibody). Analysis of non-blood samples such as bone marrow and malignant pleural effusion showed efficient infiltration of CAR-T cells into these compartments without apparent differences in their immunophenotype. This data suggests that undetectable or very low expansion of CAR-T cells in the blood might falsely indicate a treatment failure as the majority of expanding CAR-Ts *in vivo* might be localized in tumor or other tissues. In summary, our results suggest that the outcome of CAR-T cell therapy largely depends on the biological characteristics of the tumors rather than on the immunophenotype of produced CAR-T cells.

## Methods

### Patient samples

Samples were acquired from patients diagnosed with DLBCL and B-ALL who were treated with tisagenlecleucel at the Institute of Hematology and Blood Transfusion, General University Hospital in Prague, and University Hospital in Motol. All patients (or their parents/guardians) signed informed consent form. Following samples were collected from each patient: 1) apheresis used for CAR-T cell manufacturing, 2) CAR-T-cell product from infusion bag, 3) whole blood sample from patients following CAR-T infusion at three time points—first early after administration (days 2–4), second at the time of expected maximal CAR-T-cell expansion (days 10–14), and third after the retraction of the immune response (days 30–60). The control samples were obtained from age-matched healthy volunteers. In addition to FACS analysis, a total blood cell count was determined as part of a regular medical examination. Peripheral blood mononuclear cells (PBMCs) were isolated from whole blood samples by gradient centrifugation using Ficoll-Paque premium (GE Healthcare). After isolation, PBMCs were resuspended in PBS and were either stained immediately or frozen in CryoStor CS10 (StemCell Technologies) for further processing. For unfreezing, cells were thawed and cultivated in cell culture media overnight. For all experiments, CellGro media (CellGenix, Germany) supplemented with 10% heat-inactivated fetal calf serum (Gibco, United States) was used with addition of antibiotics penicillin and streptomycin (Gibco, United States).

### Patients’ characteristics

The study group includes patients with pediatric B-ALL (*n* = 5, age 3–10), adult B-ALL (*n* = 2, age 25–27), or with DLBCL (*n* = 25, age 34–77) who were eligible for treatment with tisa-cel (Table 1). In total, 32 patients received the treatment under non-clinical trial settings according to recommended clinical practice. All patients with B-ALL were in complete remission before infusion of CAR-T cells. In contrast, 24 out of 25 (96%) of the remaining patients with DLBCL had active disease with a significant tumor burden. After administration of CAR-T cells, the patients were monitored, and when necessary, treated for CRS/neurotoxicity symptoms with corticosteroids or tocilizumab according to recommended CAR-T cells’ medication protocols. A grade 2 CRS was observed in 5 patients, which required hemodynamic support. However, no patient required mechanical ventilation, all rapidly recovered from CRS symptoms. The effects of CAR-T cell therapy were determined at three and six-months’ time points *via* PET/CT (18) and by flow cytometry. Those patients who either progressed or failed to achieve at least a PR at 3 months were classified as non-responders. The remaining individuals were classified as responders and were further followed. All relevant pretreatment and post-treatment parameters are presented in Table 1. Patient P28 had a significant CAR-T expansion, so we included them as a responder; however, this patient died within a month due to complications connected with primary disease. In summary, the patients’ responses to CAR-T cell treatment and associated complications were similar to the currently known clinical experience with tisa-cel (19).

### Antibody panels and staining

Approximately five million freshly isolated PBMCs per sample were used for staining. For the first eight patients, we used PE-Labeled Human CD19 (20–291) Protein (Acro Biosystems). Later, anti-FMC63-FITC antibody by Acro Biosystems was used. Comparison of staining with protein CD19 and anti-FMC63 antibody is shown in Supplementary Figure S1. Besides detection of CAR-T cells the samples were stained with antibodies against antigens CD3, CD4, CD8, CD45RA, CD62L, CD27, CD28, CD57, PD-1, TIM-3, and TIGIT. A second detection panel against antigens CD3, CD4, CD8, CD14, CD16, CD19, CD45, CD56, and TCRgd was used to determine significant leukocyte subsets in the samples. Antibody panels used in this study were used previously (20) and can be found in Supplementary Tables S1, S2. All antibodies were titrated before use, and fluorescence-minus-one controls for selected antibodies were measured. Firstly, PBMCs were stained using a fixable blue dead cell stain kit (Thermo Fisher Scientific, United States), washed with FACS buffer, and then stained with antibody mix in Brilliant Stain Buffer (BD) at room temperature for 30 min. The samples were washed with FACS buffer prior to measurement.

### Flow cytometry

Sample data were acquired on a five-laser BD LSRFortessa instrument (BD Biosciences). All measurements were standardized using 8-peak Rainbow beads (Spherotech, Lake Forest, IL). BD CompBeads (BD, anti-rat #552844, and anti-mouse #552843) were used for compensation.

### Data analysis

Manual analysis of cytometry data was performed using FlowJo software (TreeStar).

Statistical analysis was carried out using GraphPad Prism version 9 (GraphPad Software) and MATLAB (R2022b, The MathWorks Inc.).

### Key Clinical Message

Non-responders to therapy with CD19-specific CAR-T cells (tisagenlecleucel) are characterized by undetectable expansion of CAR-T cells and in the majority of cases are diagnosed with primary refractory aggressive B-cell lymphomas..

## Data Availability

The raw data supporting the conclusion of this article will be made available by the authors, without undue reservation.
