# Supplementary material for: Characterization of the input material quality for the production of tisagenlecleucel by multiparameter flow cytometry and its relation to the clinical outcome
Source: Pathol Oncol Res. 2023 Apr 20;29:1610914. doi: 10.3389/pore.2023.1610914 (PMC10156917; doi:10.3389/pore.2023.1610914)
Supplement: Supplementary file 1 [file DataSheet1.pdf]

Supplementary table 1

Antibody panel 1

Vendor	Cat#	Antigen	Flurochrome	Clone	Dilution
BD	566460	PD1	BB700	EH12.1	50
Acro Biosystems	FM3-FY45	CAR	FITC	Y45	50
BD	565491	CD3	BV786	UCHT1	50
BD	563808	CD62L	BV650	DREG-56	50
BD	746771	Tim3	BV480	7D3	50
BD	747844	TIGIT	BV421	741182	50
BD	612846	CD45RA	BUV737	HI100	50
BioLegend	302926	CD28	PE-Cy7	CD28.2	50
eBiosciences	61-2239-42	LAG3	PE-eFluor610	3DS223H	40
Exbio	1P-158-T100	CD57	PE	TB01	160
Exbio	T4-308-T100	CD27	APC-Cy7	LT27	25
Exbio	A7-207-T100	CD8	AF700	MEM-31	50
BioLegend	300514	CD4	APC	RPA-T4	50
Invitrogen	L34962	LIVE/DEAD Fixable Blue Dead Cell Stain			

Supplementary table 2

Antibody panel 2

Vendor	Cat#	Antigen	Flurochrome
Exbio custom-made dry reagent	ED7284-1	CD16	FITC
	ED7257-1	CD56	FITC
	ED7507-1	CD14	PerCP-Cy™5.5
	ED7625-1	TCRgd	PE-Cy™7
	ED7133-1	CD19	PE-Cy™7
	ED7162-1	CD3	APC
	ED7140-1	CD4	Pacific Blue
	ED7094-1	CD45	Pacific Orange™
	ED7109-1	CD8	APC-Cy™7
Invitrogen	L34962	LIVE/DEAD Fixable Blue Dead Cell Stain	

Fig. 1S

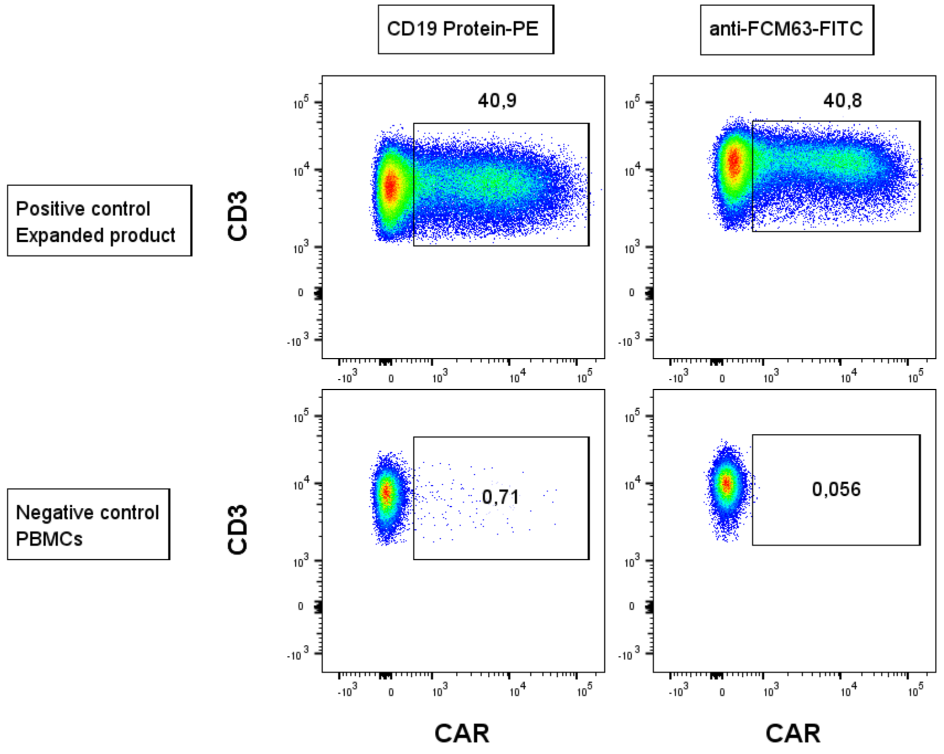

Fig. 1S Comparison between staining with PE-labeled recombinant CD19 protein and FITC-labeled anti-FMC63 antibody, both manufactured by Acro biosystems (catalog numbers CD9-HP254 and FM3-FY45 respectively). As a CAR+ sample, cells from CAR-T product expanded in a cell culture media were used.

Fig 2S

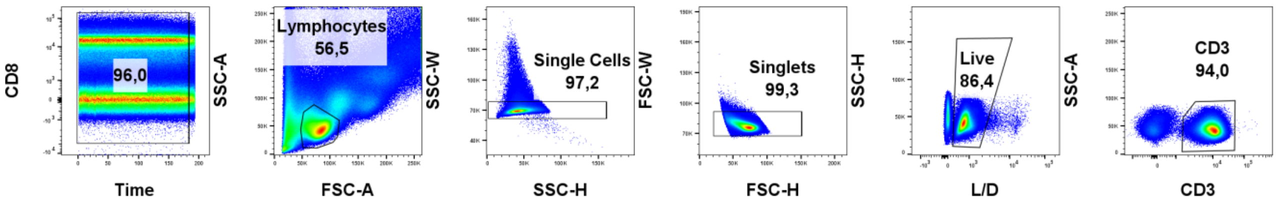

Fig. 2S Pre-gating on CD3+ cells for the phenotype analysis. First, sample integrity was checked on time scale, then lymphocytes were selected, following with single cell gates, live gate and CD3 gate.

Fig. 3S

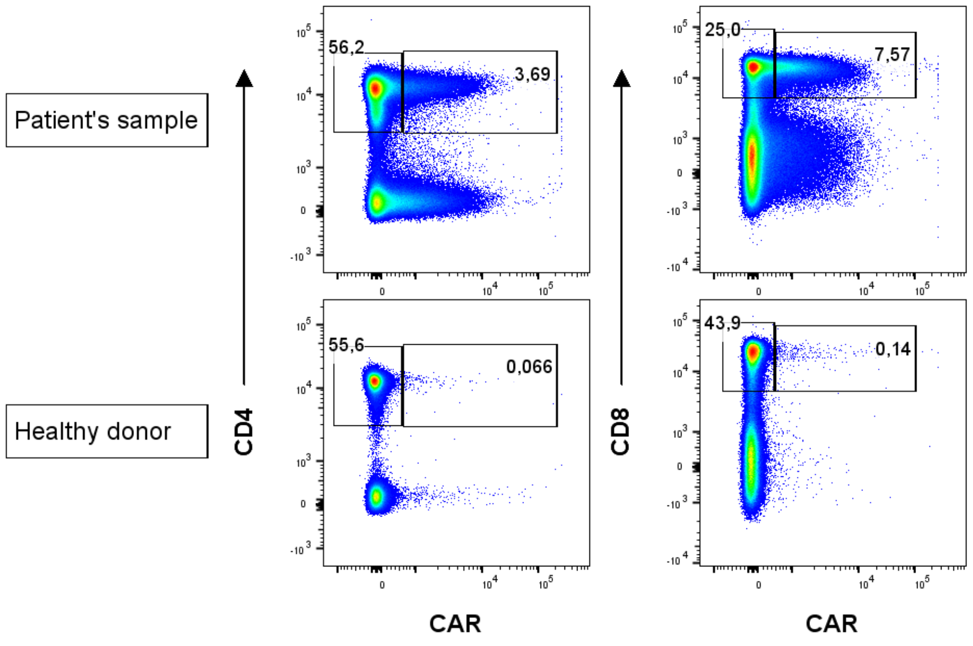

Fig. 3S Gating for CAR-T cells out of CD3+ cells. CAR+ population was determined by using a negative control – healthy donor's sample. Percentages in gates correspond to CAR+ and CAR− populations.

Fig. 4S

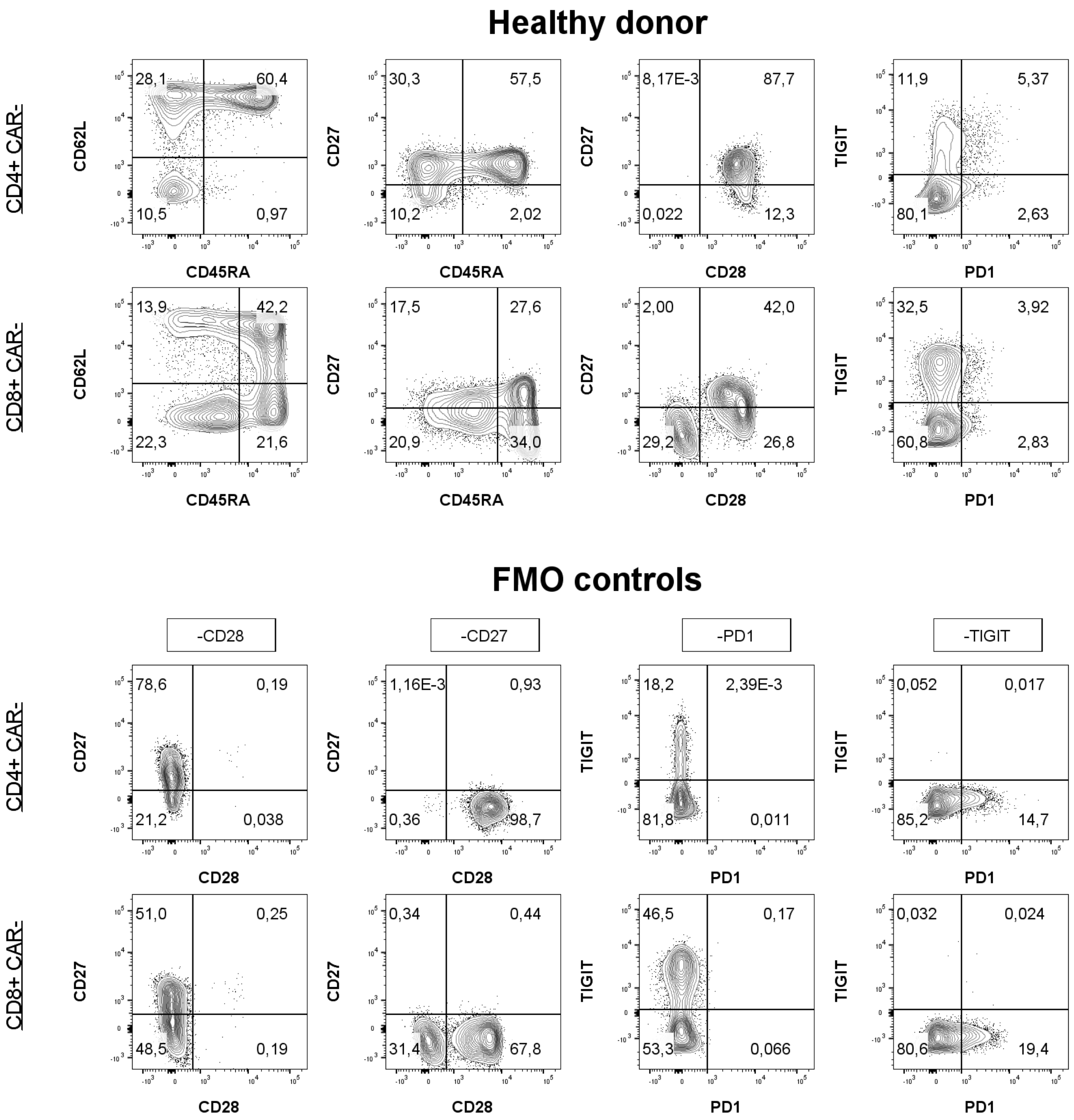

Fig. 4S Gating of T-cell phenotype. CD45RA and CD62L were gated according to healthy donor samples, FMO controls were not reliable for CD8+ cells due to their heterogenic population distribution. CD27, CD28, PD1, and TIGIT were gated according to FMO controls. Percentages are of CD4+ or CD8+ CAR− cells.

Fig. 5S

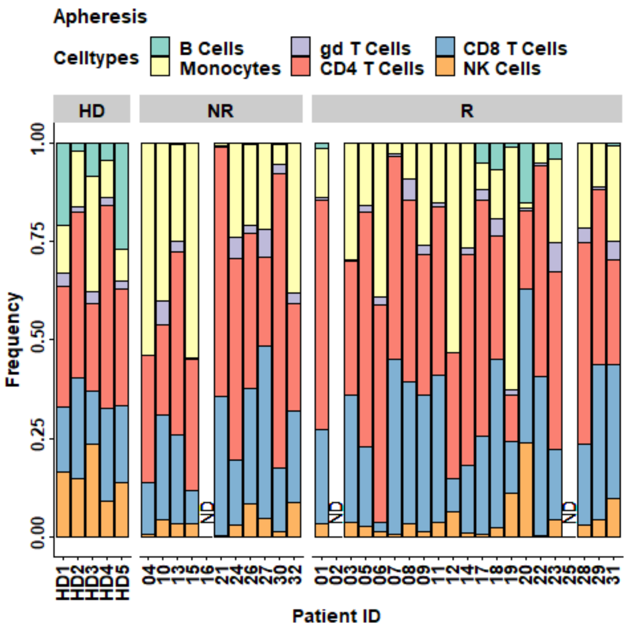

Fig. 5S Composition of leukocyte subsets in apheretic material in individual patients. HD – healthy donors, NR – non-responders, R – responders, ND – not done.

Fig. 6S

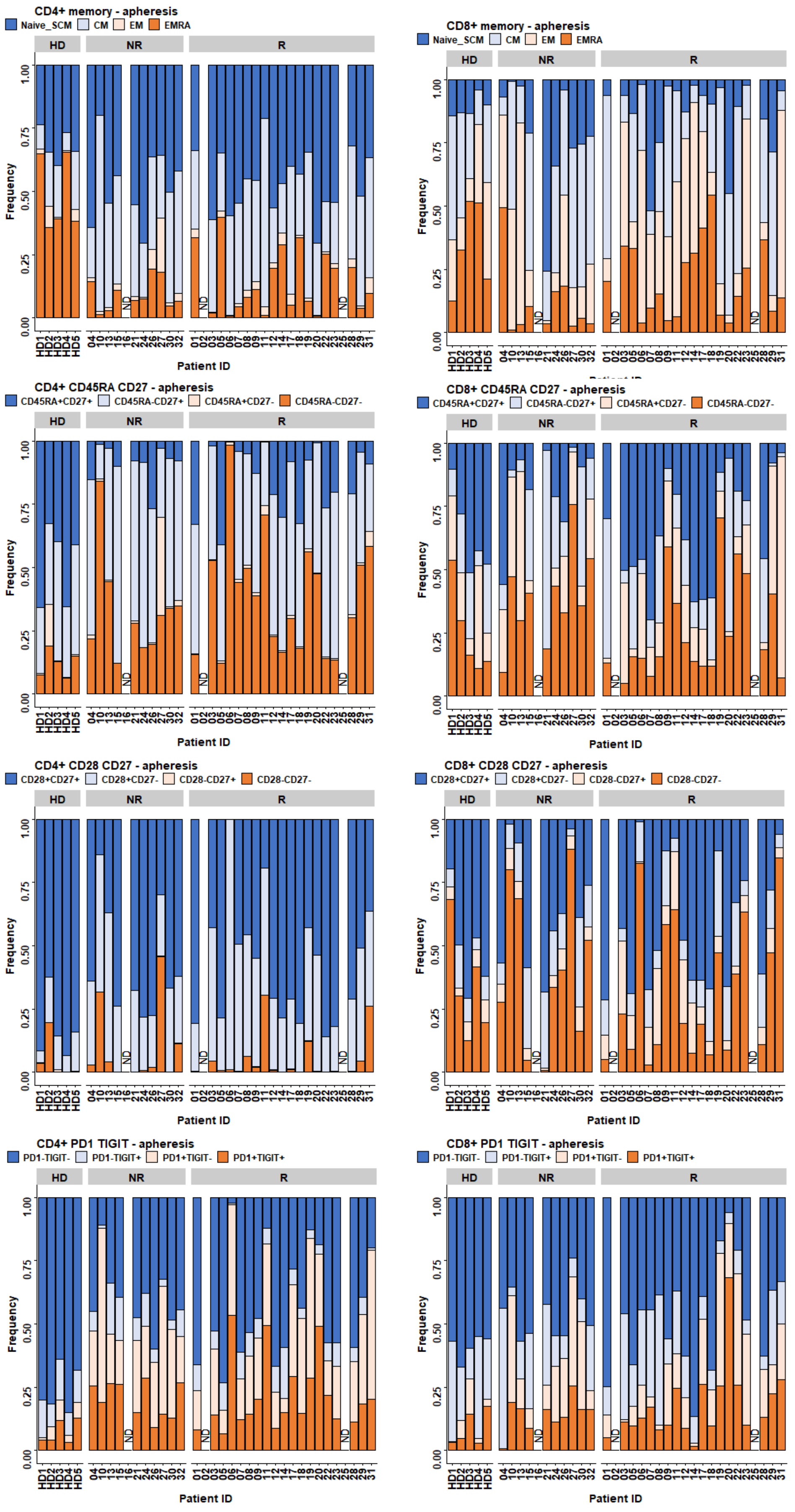

Fig. 6S Immunophenotypes of CD4+ and CD8+ T cells in apheresis of individual patients. HD – healthy donors, NR – non-responders, R – responders, ND – not done.

Fig. 7S

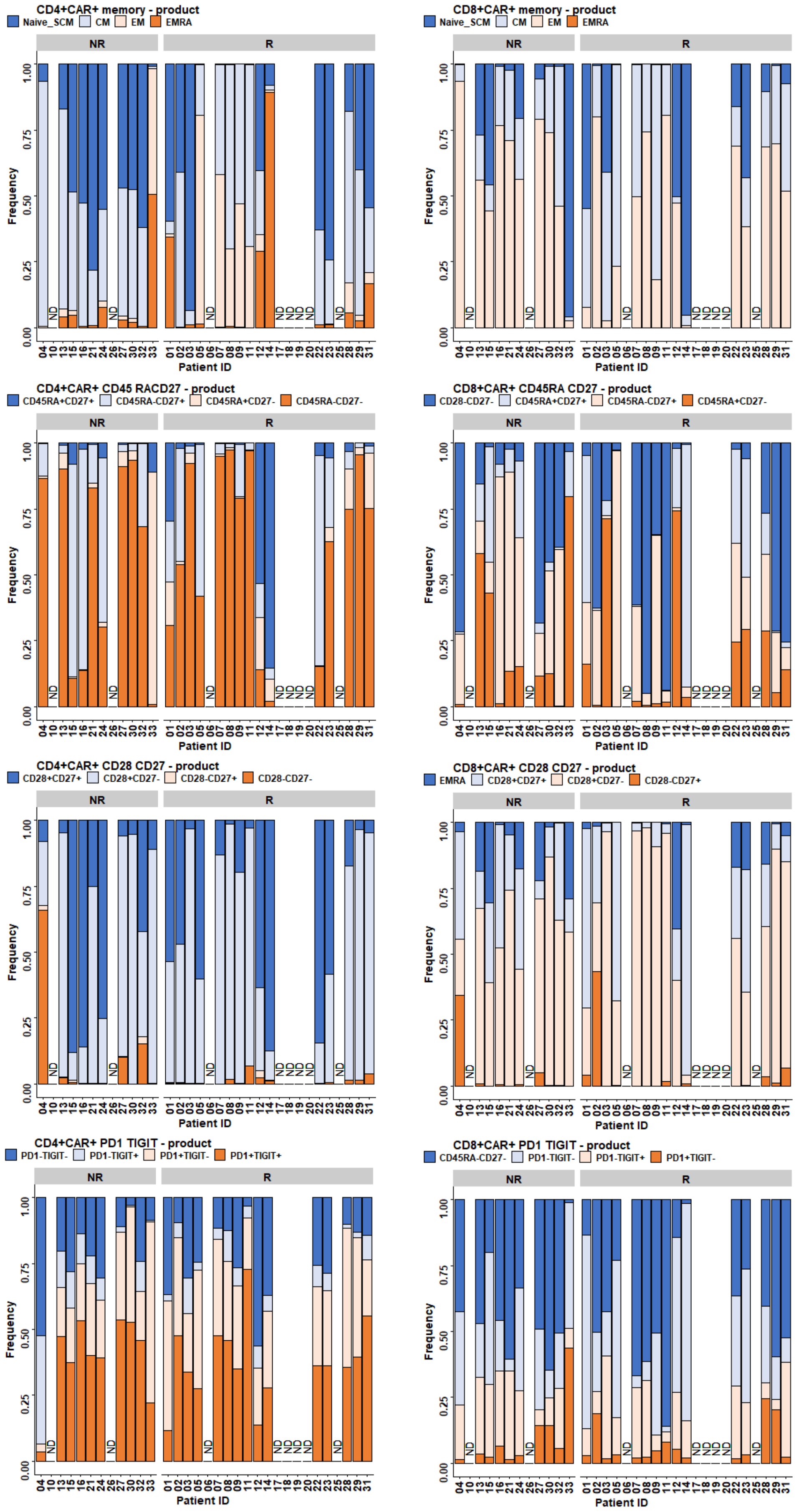

Fig. 7S Immunophenotypes of CD4+CAR+ and CD8+CAR+ T cells in the product for individual patients. NR – non-responders, R – responders, ND – not done.

Fig 8S

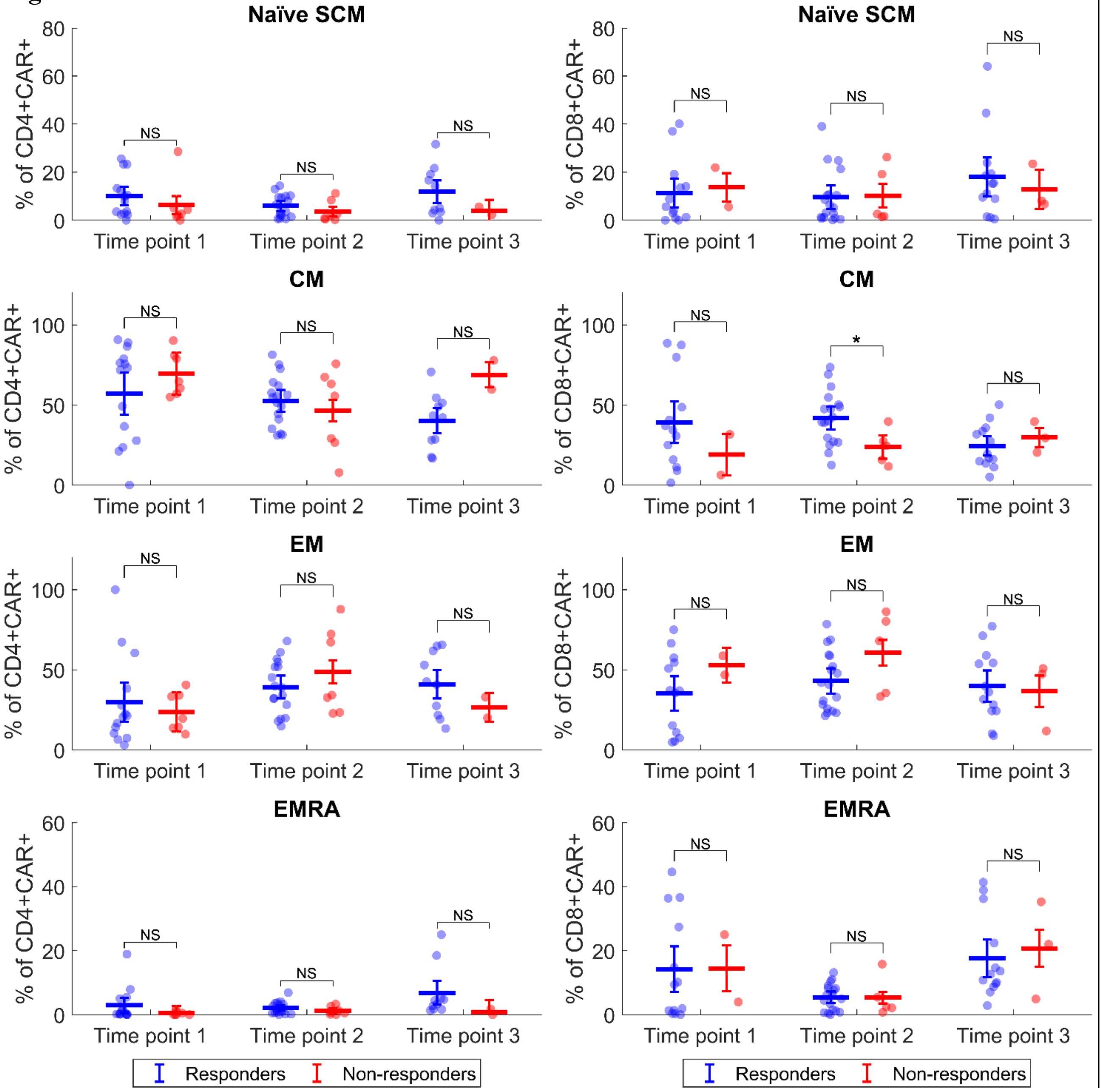
Naïve SCM
% of CD4+CAR+
% of CD8+CAR+
CM
EM
EMRA
Time point 1
Time point 2
Time point 3
NS
*
Responders
Non-responders

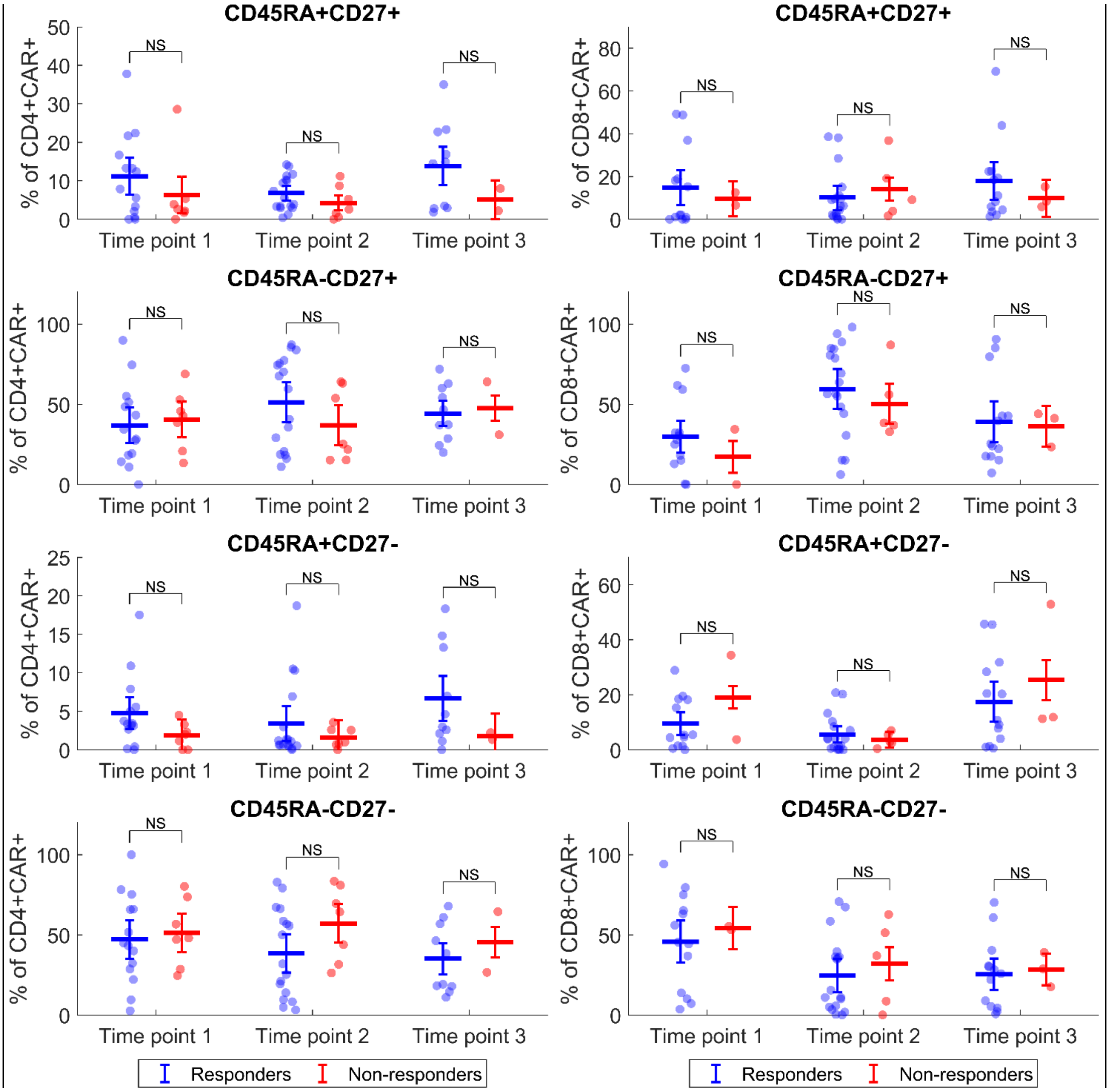
CD45RA+CD27+
% of CD4+CAR+
% of CD8+CAR+
CD45RA-CD27+
CD45RA+CD27-
CD45RA-CD27-
NS
Time point 1
Time point 2
Time point 3
Responders
Non-responders

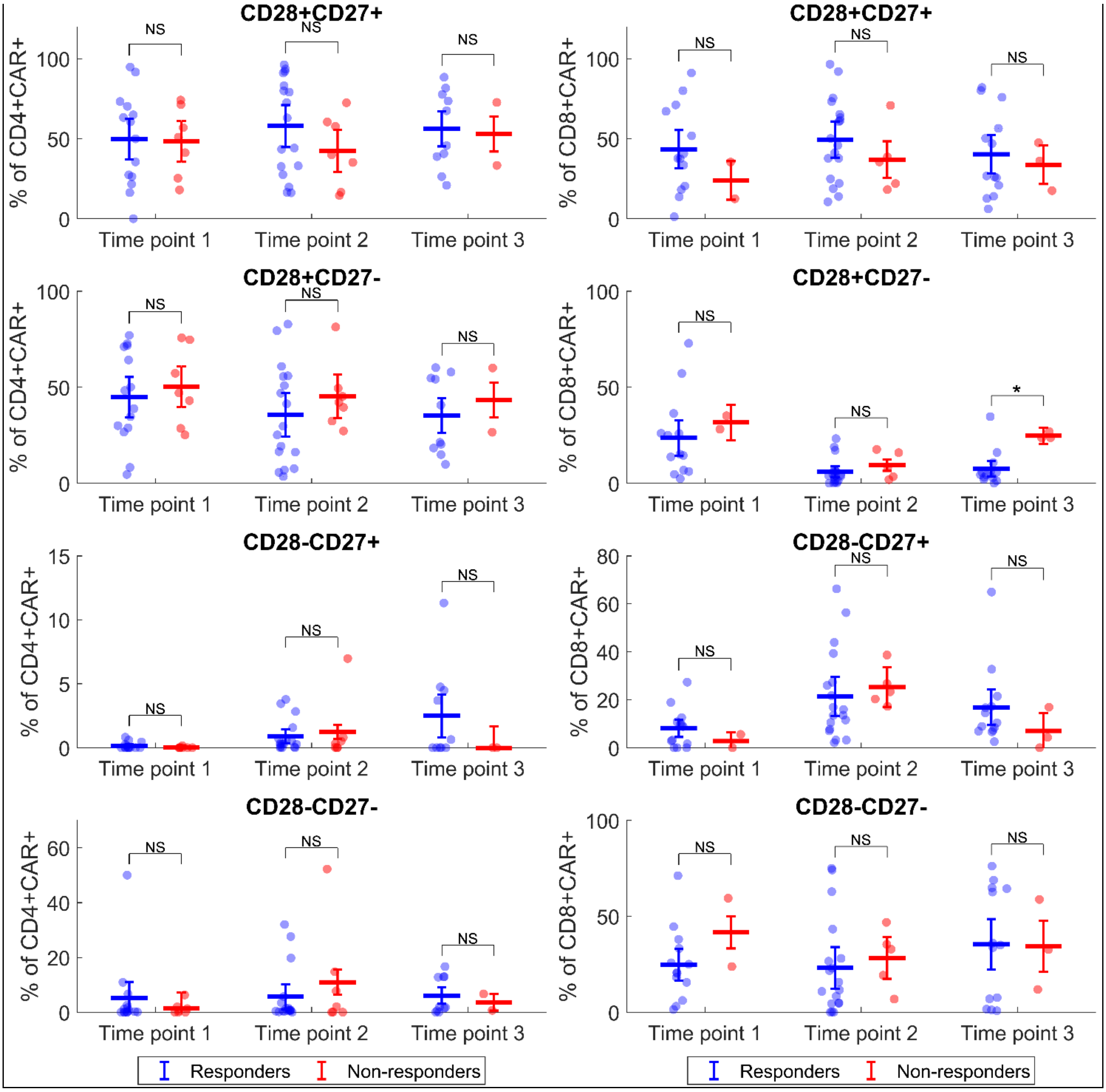

CD28+CD27+
% of CD4+CAR+
% of CD8+CAR+
CD28+CD27-
CD28-CD27+
CD28-CD27-
Time point 1
Time point 2
Time point 3
NS
*
Responders
Non-responders

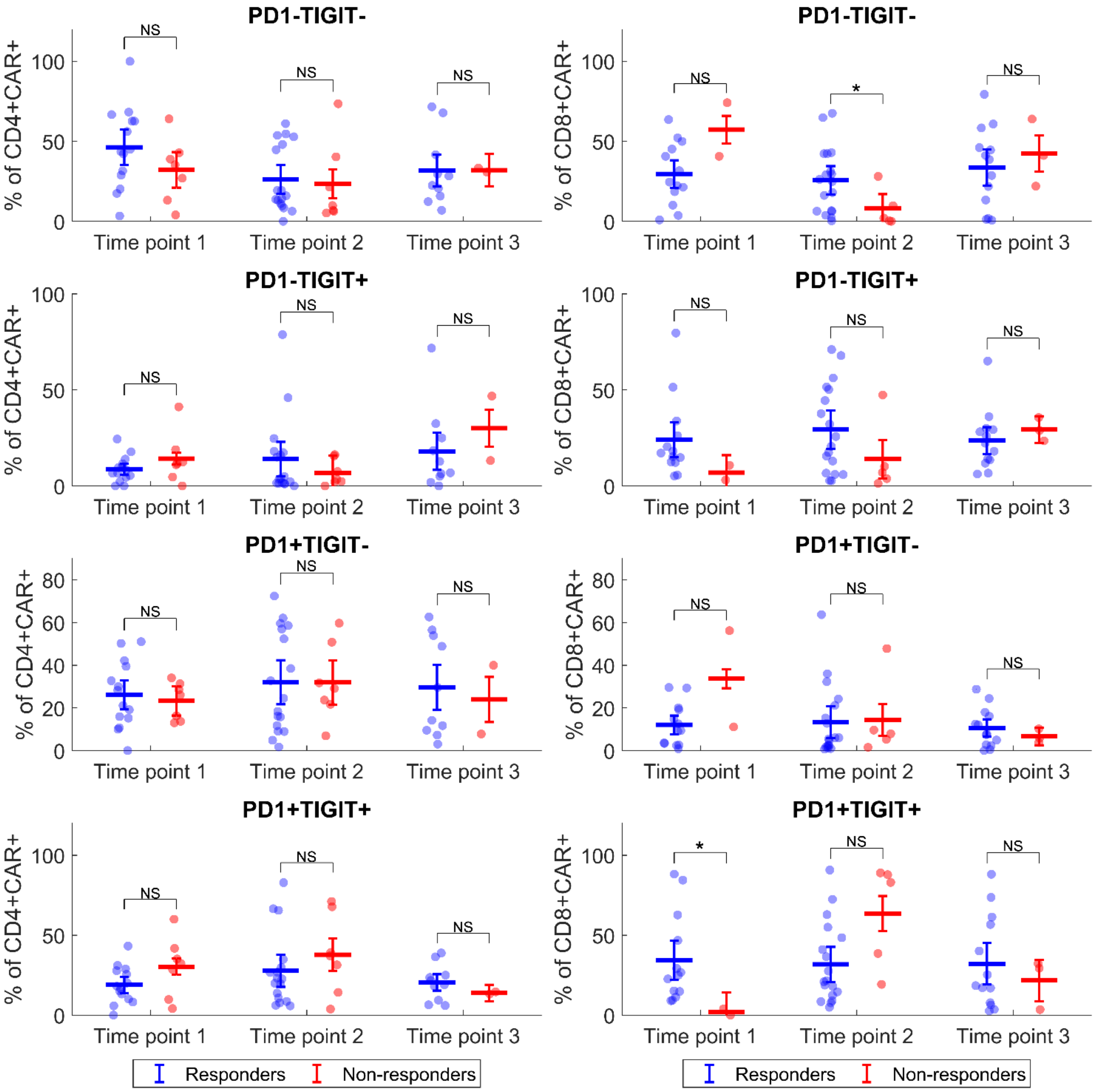

Fig. 8S Immunophenotype of CAR-T cells detected in blood at three time points in responders and non-responders. PD1+TIGIT+ percentage of CD8+CAR+ at time point 1 was higher in responders (p = 0.048), however, there were only two samples with detectable CAR-T cells in the non-responders group. Mann-Whitney test, ns - not significant, * p<0.05.

Fig. 9S

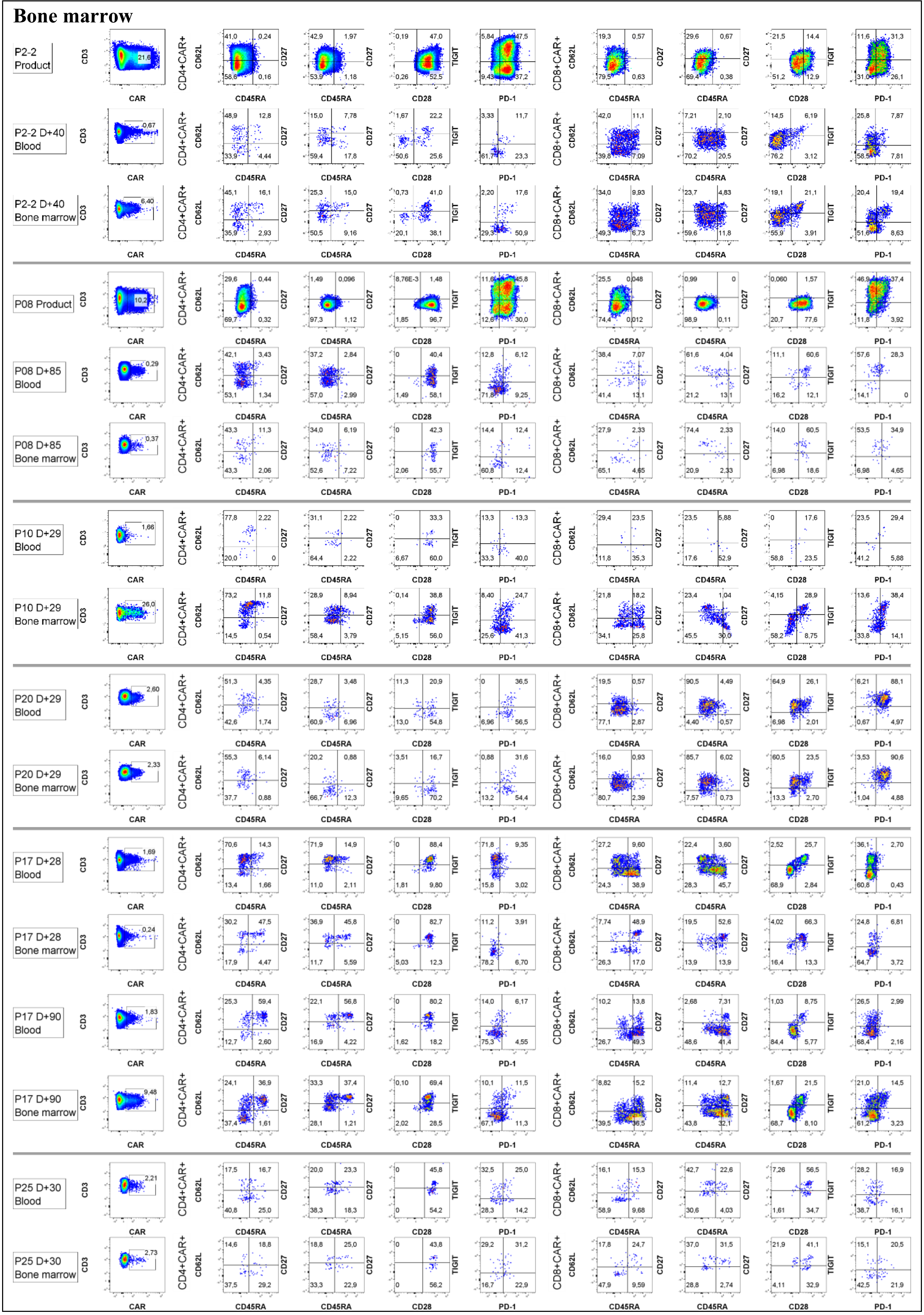
Bone marrow
P2-2 Product
P2-2 D+40 Blood
P2-2 D+40 Bone marrow
P08 Product
P08 D+85 Blood
P08 D+85 Bone marrow
P10 D+29 Blood
P10 D+29 Bone marrow
P20 D+29 Blood
P20 D+29 Bone marrow
P17 D+28 Blood
P17 D+28 Bone marrow
P17 D+90 Blood
P17 D+90 Bone marrow
P25 D+30 Blood
P25 D+30 Bone marrow
CD3
CAR
CD4+CAR+
CD8+CAR+
CD62L
CD45RA
CD27
CD28
TIGIT
PD-1

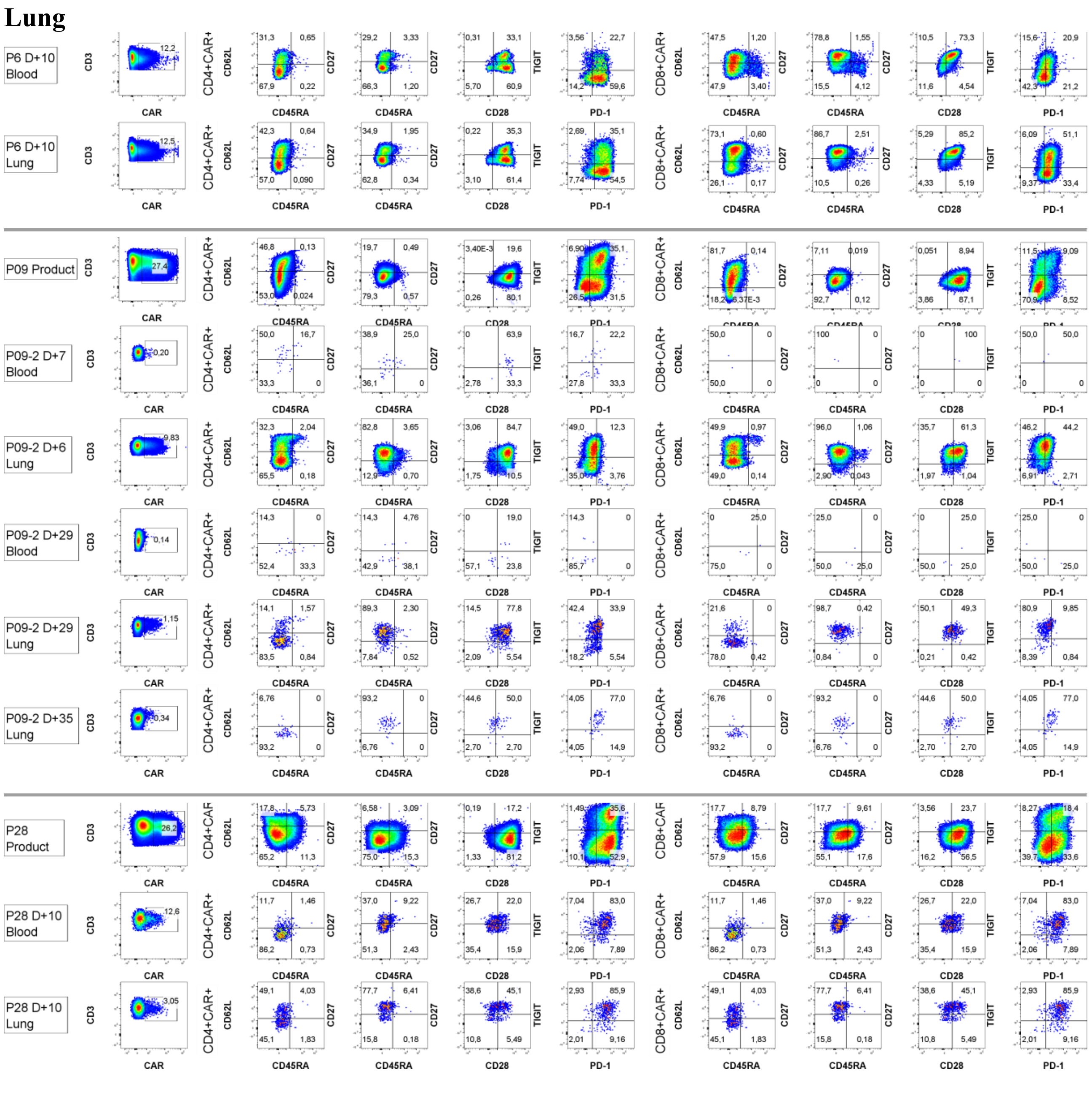

Fig 9S Phenotypes of all bone marrow and lung samples. Only samples with more than 0.1% of CD4+CAR+ or CD8+CAR+ cells are shown. Data for available products are presented for comparison.
